# Modulational instability and associated multiple dark solitons in relativistically degenerate electron-positron-ion plasmas

**DOI:** 10.1038/s41598-025-10607-9

**Published:** 2025-09-30

**Authors:** R. Jahangir, W. Masood, M. Siddiq, N. Batool, Shakir Ullah, Hanan Al-Ghamdi, C. G. L. Tiofack, Samir A. El-Tantawy

**Affiliations:** 1 National Centre for Physics, Shahdara Valley Road, Islamabad, 44000 Pakistan; 2 COMSATS University Islamabad, Park Road, Chak Shahzad, Islamabad, 44000 Pakistan; 3 Government Post Graduate College Karak, Karak, 27200 Pakistan; 4 Department of Physics, College of Science, Princess Nourah bint Abdulrahman University, P.O. Box 84428, Riyadh, 11671 Saudi Arabia; 5 Faculty of Sciences, University of Maroua, P.O. Box 814, Maroua, Cameroon; 6 Department of Physics, Faculty of Science, Port Said University, Port Said, 42521 Egypt; 7 Department of Physics, Faculty of Science, Al-Baha University, P.O. Box 1988, Al-Baha, Saudi Arabia

**Keywords:** Electron-positron-ion plasmas, Relativistically degenerate electrons and positrons, Modulational instability and nonlinear Schrödinger equation, Hirota method, Multiple dark solitons, Fluid dynamics, Plasma physics

## Abstract

The modulational instability (MI) of the ion-acoustic waves (IAWs) is analyzed in an unmagnetized electron-positron-ion (EPI) plasma having relativistically degenerate electrons and positrons. For this purpose, the nonlinear Schrödinger (NLS) equation is derived using the derivative expansion method. The criteria for MI are numerically examined in the vicinity of pulsars, and it is observed that for both nonrelativistic and ultrarelativistic regimes, the EPI plasma remains modulationally stable. The nonlinear structures, derived using Hirota’s method, are the dark envelope solitons. The dark solitons are further classified as black and gray solitons depending on the relation between the amplitude of the vacuum wave train and the propagation vector of the envelope soliton. The increase in the positron concentration is observed to stabilize the EPI system and is also found to reduce the amplitude of the dark envelope soliton. The interaction of black and gray, as well as the two gray solitons, shows that, being nonlinear waves, the condition of linear superposition is not met at the interaction point for dark envelope solitons. Furthermore, the spatial regime of the soliton interaction is reduced for the gray-gray envelope interaction compared to the black-gray envelope interaction, as well as the ultrarelativistic case compared to the nonrelativistic case. This study is novel as it discusses the interaction of dark solitons of the NLS in the context of plasmas for the first time. The results of the present model are beneficial to comprehend the dark envelope soliton interaction for astrophysical plasmas; however, by incorporating changes in the behavior of electrons and positrons, they are relevant for diverse conditions in laboratory and space plasmas.

## Introduction

Electron-positron (EP) plasmas are rich in nature and have been found to exist in neutron stars, pulsars, black holes, active galactic nuclei (AGNs), accretion disks, and the electrosphere of strange-quark stars is also conjectured to exist in the early universe^1–4^. Shortly after the Big Bang, it is conjectured that megaelectronvolt temperatures were present for a second, and the universe was packed with EP plasmas. The loss of energy in low-luminosity active galactic nuclei (LLAGN) is presumed to come from inside in the shape of teraelectronvolt radiation, which could possibly create EP pairs^5^.There is a constant balance between the thermal energy due to nuclear reactions occurring inside the star and the gravitational pull. At the end of its life, the loss of thermal energy makes the star collapse due to gravitational pull. Until a certain point, the degeneracy pressure resists collapse. Owing to the collapse, the temperature and the density become enormously high, and the constituent particles accelerate either by the electromagnetic field or the gravitational collapse. The resulting collision of the particles can also create EP pairs both in white dwarfs and neutron stars^6^. EP pairs are also surmised to be formed by the presence of strong magnetic fields near the polar gaps of the pulsars. Initially, gamma rays are produced due to the acceleration of electrons in the presence of gigantic B fields through a phenomenon called curvature radiation, and subsequently, the gamma rays produce EP pairs. It is hypothesized that ions exist in this environment, potentially originating within the core of compact stars or entering from external sources via an accretion process^7^.The uniqueness of EP plasmas lies in the fact that we have two species with equal masses but opposite charges. It essentially means that we do not have a different timescale, unlike an ordinary electron-ion plasma. Such a situation quickly changes in the presence of ions, and both slow and fast dynamics can then be studied, corresponding to the ion and EP time scales, respectively. Over the past several decades, a substantial body of research has investigated electrostatic and electromagnetic behaviors in electron-positron-ion (EPI) plasmas^8–11^. The study of quantum effects becomes important when the de Broglie wavelength of the particles becomes comparable to the interparticle distance. Enhancing the concentration of the particles makes this happen and, therefore, dense systems are usually quantum systems^12^. Note that in an EPI plasma, electrons and positrons are treated quantum mechanically, while ions are considered to be classical, owing to their three orders of magnitude higher mass than them. Such a model is typically referred to as the quantum hydrodynamic (QHD) model. In recent years, extensive research has been conducted to examine both linear and nonlinear wave modes in quantum plasmas^8,13–15^. The propagation of ion-acoustic waves in relativistically degenerate plasmas has also emerged as an active and important area of research^16,17^. Solitons are special due to their particle-like characteristics that occur as a consequence of the balance between dispersion and weak nonlinearity in a system^18^. Multisolitons play an important role in the transmission of momentum and energy within a system, due to their robustness property during propagation and interaction^19,20^. Inverse scattering usually achieves these solutions^21^, however, the applicability of the technique is a bit demanding for some practical problems. On the other hand, the Hirota method, introduced in 1971 by Hirota^22^ for the Korteweg-de Vries (KdV )equation, is efficient for multisoliton solutions, since it converts the problem from analytic to algebraic^23^. Many researchers have used the method to study multisoliton solutions of several integrable nonlinear partial differential equations (NLPDEs)^24–28^.Nonlinear Schrödinger (NLS) equation, being the nonlinear extension of the Schrödinger equation, has a broad spectrum of applications in various areas such as plasmas, fluid dynamics, electromagnetism, deep water waves, and many more. In particular, it is helpful for the self-focusing of optical pulses in optical fiber communication^29^. The modulated waves propagate in these systems due to the competing effects of group dispersion and nonlinearity in the medium. The analysis of modulational instability (MI) is carried out with the help of a monochromatic solution of the NLS equation. Envelope solitons are one of the localized modulated solitary wave solutions of the NLS equation, further divided into bright and dark solitons, depending on the MI of the wave in the medium^30–32^. The dark envelope solitons appear as a local dip or a concavity on the continuous background, while the bright envelope solitons result in a localized intensity peak^33^. A few researchers have explored the dynamics of envelope solitons concerning the NLS equation for ion acoustic and electron acoustic waves in plasmas^34,35^.The dark solitons, which tend to be less likely to be disrupted by background noise compared to bright solitons, occur when the system is modulationally stable. The dark solitons have been observed experimentally in optical fibers^36^, spin waves in ferromagnetic films^37^, complex plasma systems^38^, surface gravity waves in water^39^, Bose-Einstein condensates^40^, and studied theoretically for plasma systems^41,42^. The head-on collision of dark solitons in optical fibers^43,44^ and plasmas^45^, as well as the overtaking collision of dark envelope solitons in optical fibers^46^ has been examined; however, less attention is given to the overtaking interaction of dark envelope solitons in plasmas^47^. The Hirota method is employed to investigate the overtaking interaction of dark envelopes, also known as the hole envelopes of the NLS equation^48^.In this article, we examine the interaction of ion acoustic dark envelope solitons in dense degenerate EPI plasmas. Section “Plasma fluid model ” deals with the basic fluid model and the equations governing the ion acoustic waves with relativistically degenerate electrons and positrons. Section “The derivative expansion method for NLS equation” employs a derivative expansion method to derive the NLS equation. Section “Stability analysis” deals with the MI of ion-acoustic waves (IAWs) for both nonrelativistic and ultrarelativistic limits of electrons and positrons. Section “Exact dark soliton solutions” presents the dark envelope solitons obtained by using Hirota’s method and their interaction. Section “Discussion and conclusion” finally recapitulates the findings of this investigation.

## Plasma fluid model 

We assume a collisionless, homogeneous, unmagnetized, multicomponent plasma comprising ions, electrons, and positrons to study the propagation of one-dimensional IAWs in a dense plasma. We consider a semi-quantum plasma model in which ions are assumed to be classical because of their mass being much larger than that of electrons and positrons. On the other hand, electrons and positrons have opposite charges but the same mass and are assumed to be relativistically degenerate. The general condition to ignore the electron-positron annihilation time in a plasma system is that electrons and positrons must last long enough to complete a full plasma cycle before annihilating, which is met by both nonrelativistic and ultrarelativistic electrons and positrons^8^. The well-known continuity and momentum equations governing the dynamics of inertial ions in one dimension for such a system are as follows:

1 $$\begin{aligned}&\partial _{t}n_{i}+\partial _{x}\left( n_{i}u_{i}\right) =0,\end{aligned}$$

2 $$\begin{aligned}&\partial _{t}u_{i}+u_{i}\partial _{x}u_{i} =-\frac{e}{m_{i}}\partial _{x} \phi , \end{aligned}$$

along with Poisson equation for the electrostatic potential $\phi$

3 $$\begin{aligned} \partial _{x}^{2}\phi =4\pi e\left( n_{e}-n_{p}-n_{i}\right) . \end{aligned}$$

The dynamics of relativistically degenerate inertialess electrons and positrons are described by the following equations

4 $$\begin{aligned} 0&=en_{e}\partial _{x}\phi -\partial _{x}P_{Fe},\end{aligned}$$

5 $$\begin{aligned} 0&=-en_{p}\partial _{x}\phi -\partial _{x}P_{Fp}. \end{aligned}$$

Here, the physical quantities ($n_{i}$, $n_{e}$, $n_{p}$) represent, respectively, the number densities of ions, electrons, and positrons; $u_{i}$ is the speed of ion particles, $\phi$ is the electrostatic potential, and ($P_{Fe}$, $P_{Fp}$) are the relativistic Fermi pressures of electrons and positrons. At equilibrium, the charge neutrality condition reads: $n_{i0}+n_{p0}\simeq n_{e0}$ for singly charged positive ions, where the subscript “0” stands for the unperturbed quantities.

The Chandrasekhar equation of state for relativistic Fermi pressure of electrons and positrons in dense plasmas is given by^49^

6 $$\begin{aligned} P_{Fj}=\left( \frac{\pi m_{e}^{4}c^{5}}{3h^{3}}\right) \left[ (2\eta _{j}^{3}-3\eta _{j})(\eta _{j}^{2}+1)^{1/2}+3\sinh \eta _{j}^{-1}\right] , \end{aligned}$$

where the subscript “$j=(e,p)$” stands for electrons and positrons, and $\eta _{j}$ is the Chandrasekhar’s relativistic parameter related to the Fermi momentum of the electron/positron $p_{Fj}(\equiv 3h^{3}n_{j}/8\pi )^{1/3}$ as $\eta _{j}=p_{Fj}/m_{e}c=\left( n_{j}/n_{c}\right) ^{1/3}$. Here, $n_{c}\simeq 5.9\times 10^{29}cm^{-3}$ is the normalizing value. The nonrelativistic and ultrarelativistic limits of the Fermi pressure in the polytropic forms, i.e., $P_{Fj}\propto n_{j}^{5/3}$ and $P_{Fj}\propto n_{j}^{4/3}$, respectively, are recovered from above equation of state for $\eta _{j}\rightarrow 0$ and $\eta _{j}\rightarrow \infty$, respectively. The pressure may be expanded around the equilibrium number density by using Taylor’s expansion^50,51^, which gives the following expression

7 $$\begin{aligned} P_{Fj}\sim P_{j0}+\frac{2}{3\gamma _{j0}}E_{Fj}(\delta n_{j})+\frac{E_{Fj}(2+\eta _{j0}^{2})}{9n_{j0}\gamma _{j0}^{3}}(\delta n_{j})^{2} +\cdots , \end{aligned}$$

where $\delta n_{j}$ is the perturbed density and $\gamma _{j0}=\sqrt{1+\eta _{j0}^{2}}$. We may scale all variables at this step by proper quantities to get a dimensionless system of equations. For this purpose, we scale all dependent and independent variables as $n_{j}\rightarrow n_{j}/n_{j0}$, $u_{i}\rightarrow u_{i}/C_{s}$ and $\phi \rightarrow e\phi /E_{Fe}$, $P_{Fj}\rightarrow E_{Fe}n_{j0}$, $x\rightarrow \lambda _{Fe}$, and $t\rightarrow \omega _{pi}^{-1}$, where plasma frequency $\omega _{pi}=(4\pi n_{i0}e^{2}/m_{i})^{1/2}$, Fermi wavelength $\lambda _{Fe}=(E_{Fe}/4\pi n_{i0}e^{2})^{1/2}$, acoustic speed, $C_{s}=\sqrt{E_{Fe}/m_{i}},$ and Fermi energy $E_{Fe}=(\hbar ^{2})(3\pi ^{2}n_{e})^{2/3}/2m_{e}$. Consequently, the normalized set of fluid equations takes the form

8 $$\begin{aligned} & \partial _{t}n_{i}+\partial _{x}\left( n_{i}u_{i}\right) =0, \end{aligned}$$

9 $$\begin{aligned} & \partial _{t}u_{i}+u_{i}\partial _{x}u_{i}=\partial _{x}\phi , \end{aligned}$$

10 $$\begin{aligned} & \partial _{x}^{2}\phi =\mu n_{e}-\upsilon n_{p}-n_{i}, \end{aligned}$$

11 $$\begin{aligned} & \partial _{x}\phi =\frac{1}{n_{e}}\partial _{x}\left( 1+\Gamma _{1}(\delta n_{e})+\Gamma _{2}(\delta n_{e})^{2}+\cdots \right) , \end{aligned}$$

and

12 $$\begin{aligned} \partial _{x}\phi =-\alpha ^{2/3}\frac{1}{n_{p}}\partial _{x}\left( 1+\Delta _{1}(\delta n_{p})+\Delta _{2}(\delta n_{p})^{2}+\cdots \right) , \end{aligned}$$

where, $\Gamma _{1}=2/3\gamma _{e0}$, $\Gamma _{2}=(2+\eta _{e0}^{2})/9\gamma _{e0}^{3}$, $\Delta _{1}=2/3\gamma _{p0},$ and $\Delta _{2}=(2+\eta _{p0} ^{2})/9\gamma _{p0}^{3}$. For the normalized case (dimensionless form), we define the ratio $\alpha =n_{p0}/n_{e0}$, which leads to $\mu =n_{e0} /n_{i0}=1/(1-\alpha )$ and $\upsilon =n_{p0}/n_{i0}=\alpha /(1-\alpha )$, from the charge neutrality condition. Here, it is crucial to underline that the validity of our model depends on certain criteria over the temperatures,

13 $$\begin{aligned} T_{Fi}<T_{i}<T_{e}<T_{Fe}(/T_{Fp}), \end{aligned}$$

which means that the dominant temperature for dense degenerate plasmas is the Fermi temperature of electrons and positrons, where $T_{Fj}=(\hbar ^{2} )(3\pi ^{2}n_{j})^{2/3}/2m_{e}k_{B}$. Since, for dense plasmas, the Fermi temperatures are dependent upon the number densities; therefore, contrary to the essential condition $T_{e}\gg T_{i}$ for IAWs in classical plasma, the only significant parameters for ion acoustic waves in dense plasmas are the densities of electrons and positrons.

## The derivative expansion method for NLS equation

To derive the NLS equation, we use the derivative expansion method (DEM) to examine the modulation of the amplitude of IAWs propagating along the *x*-axis^52,53^. Accordingly, we introduce the stretched coordinates as follows:

14 $$\begin{aligned} \xi =\epsilon \left( x-v_{g}t\right) \ \& \tau =\epsilon ^{2} t, \end{aligned}$$

where $v_{g}$ is the group velocity of the wave packet and $\epsilon$ is a real and small parameter ($\epsilon \ll 1$), which measures the smallness of perturbation. According to the DEM, the following operators for the independent variables are considered

15 $$\begin{aligned} \partial _{x}=\partial _{x}+\epsilon \partial _{\xi } \& \partial _{t}=\partial _{t}-\epsilon v_{g}\partial _{\xi }+\epsilon ^{2}\partial _{\tau }. \end{aligned}$$

For a 1-D propagation of IAW, the modulation of the wave packet appears along the *x*-axis, which may be introduced through the phase ($kx-\omega t$). Therefore, the dependent variables are expanded as follows

16 $$\begin{aligned} A=A_{0}+ {\displaystyle \sum \limits _{m=1}^{\infty }} \epsilon ^{m} {\displaystyle \sum \limits _{l=-\infty }^{\infty }} A_{l}^{(m)}\exp (i\Theta ), \end{aligned}$$

where, $A\equiv A\left( x,t\right) =(n_{i},n_{e},n_{p},u_{i},\phi )$, $A_{0}=(1,1,1,0,0),$ $A_{l}^{(m)}\equiv A_{l}^{(m)}(\xi ,\tau )$, $\Theta =l(kx-\omega t)$, and $i=\sqrt{-1}$. Here, *A* satisfies the reality condition, $A_{-l}^{(m)}=A_{l}^{(m)*}$ and the asterisk represents the complex conjugate. We get a set of reduced equations by employing the stretching (15) and expansion (16) in Eqs. (8)–(12) and collecting the various powers of $\epsilon$. For lowest-order ($m=1$ & $l=1$), we get the expressions in terms of $\phi _{1}^{1}$ for which the first harmonic solution becomes

17 $$\begin{aligned}&n_{1}^{(1)}=\frac{k}{\omega }\text u_{1}^{(1)}=\frac{k^{2}}{\omega ^{2} }\phi _{1}^{(1)},\nonumber \\&n_{e1}^{(1)}=\frac{1}{\Gamma _{1}}\phi _{1}^{(1)},\text n_{p1}^{(1)} =\frac{-1}{\alpha ^{2/3}\Delta _{1}}\phi _{1}^{(1)},\nonumber \\&\left. n_{1}^{(1)}-k^{2}\phi _{1}^{(1)}-\mu n_{e1}^{(1)}-\upsilon n_{p1}^{(1)}=0.\right. \end{aligned}$$

By solving system (17), the following dispersion relation is obtained

18 $$\begin{aligned} \frac{\omega ^{2}}{k^{2}}=\frac{1}{k^{2}+c_{1}}, \end{aligned}$$

with

$$\begin{aligned} c_{1}=\frac{1}{\Gamma _{1}(1-\alpha )}+\frac{\alpha ^{1/3}}{\Delta _{1}(1-\alpha )}. \end{aligned}$$

It is evident from Eq. (18) that the modified dispersion relation of the IAW now varies with the densities of electrons and positrons for the relativistically degenerate EPI plasma. The obtained dispersion relation agrees well with the results of Rehman et al.^15^. In the long-wavelength limit, it reduces to $\omega /k=v_{ph}=1/\sqrt{c_{1}}$, which is the modified normalized phase velocity of the IAWs in EPI plasmas.

For the second order $m=2$ and the first harmonic $l=1$, we get the quantities in terms of the first-order potential $\phi _{1}^{(1)}$ which provide the following compatibility condition.

19 $$\begin{aligned} v_{g}=\partial _{k}\omega =\frac{\omega }{k}\left( 1-\omega ^{2}\right) . \end{aligned}$$

The second order also gives the equations for constant harmonic terms $\left( A_{0}^{(2)}\right)$ and second harmonic terms $\left( A_{2}^{(2)}\right)$ of the carrier wave in terms of the $\phi _{1}^{(1)}$. These equations, along with the third-order and first-harmonic equations ($m=3$, $l=1$) may be solved simultaneously to obtain the following NLS equation

20 $$\begin{aligned} i\partial _{\tau }\Psi +P\partial _{\xi }^{2}\Psi +Q\Psi \left| \Psi \right| ^{2}=0, \end{aligned}$$

where, we have replaced $\phi _{1}^{\left( 1\right) }$ by $\Psi$ for the sake of simplicity and the coefficients of the dispersion *P* and nonlinearity *Q* are, respectively, defined as

21 $$\begin{aligned} P=-\frac{3\omega ^{3}}{2k^{2}}(1-\omega ^{2}), \end{aligned}$$

and

22 $$\begin{aligned} Q=-\frac{\omega ^{3}}{2k^{2}}\left( \begin{array}{c} \frac{k^{6}}{\omega ^{6}}C_{0}-\frac{L_{1}}{2\Gamma _{1}}(1-\frac{6\Gamma _{2} }{\Gamma _{1}})-\frac{M_{1}}{2\alpha \Delta _{1}}(1-\frac{6\Delta _{2}}{\Delta _{1}})\\ +L_{02}\left( C_{1}-L_{1}+\frac{M_{1}}{\alpha ^{1/3}}\right) +L_{22}\left( C_{2}-L_{1}+\frac{M_{1}}{\alpha ^{1/3}}\right) \end{array} \right) , \end{aligned}$$

with

$$\begin{aligned} L_{1}&=\frac{\left( 1-2\frac{\Gamma _{2}}{\Gamma _{1}}\right) }{(1-\alpha )\Gamma _{1}^{2}},M_{1}=\frac{\left( 1-\frac{2\Delta _{2}}{\Delta _{1}}\right) }{(1-\alpha )\Delta _{1}^{2}},\\ C_{0}&=\frac{(5\omega ^{4}+15-18\omega ^{2})}{2(1-\omega ^{2})^{2}} ,C_{1}=\frac{3k^{4}}{\omega ^{4}},C_{2}=\frac{k^{4}(3-2\omega ^{2})}{\omega ^{4}(1-\omega ^{2})^{2}},\\ L_{02}&=\frac{\left[ k^{2}(-3+2\omega ^{2})+L_{1}\omega ^{2}v_{g}^{2} -M_{1}\omega ^{2}v_{g}^{2}\alpha ^{-1/3}\right] }{\left[ \omega ^{2}-k^{2} v_{g}^{2}(1-\omega ^{2})\right] },\\ L_{22}&=\frac{\left( C_{1}-L_{1}-\alpha ^{-1/3}M_{1}\right) }{6k^{2}}. \end{aligned}$$

The group dispersion *P* is related to the dispersion $\omega$ by the relation $P=\frac{1}{2}(\partial _{k}^{2}\omega )$, while the nonlinearity *Q* arises due to the interaction of carrier waves in the background plasma. It may be seen from the expressions of the dispersion (21) and nonlinearity (22) coefficients that they depend on the wave number *k* and the positron concentration $\alpha$. These coefficients actually determine the modulationally stable/unstable regimes of the IAWs in relativistic EPI plasmas.

## Stability analysis

The MI of IAWs in relativistic EPI plasma is studied by assuming a monochromatic solution $\Psi =\left( \Psi _{0}+\delta \Psi \left( \acute{k} \xi -\acute{\omega }\tau \right) \right) e^{i\Delta \tau }$ of the NLS Eq. (20), where $\delta \Psi$ is a small perturbation, $\Psi _{0}$ is the amplitude of the carrier wave with $\Psi _{0}\gg \left| \delta \Psi \right|$, $\Delta$ is the nonlinear frequency shift, $0<\acute{k}\ll k$ is the modulated wave number, and $\acute{\omega }\ll \omega$ is the frequency of the modulation. Using the standard procedure adopted by Amin et al.^54^, the nonlinear dispersion relation for the modulated IAW may be given as

23 $$\begin{aligned} \acute{\omega }^{2}=(P\acute{k})^{2}\left[ \acute{k}^{2}-k_{c}^{2}\right] , \end{aligned}$$

with the following critical propagation vector

$$\begin{aligned} k_{c}=\sqrt{\frac{2Q\Psi _{0}^{2}}{P}}. \end{aligned}$$

Remember that $\acute{\omega }$ becomes real for $k_{c}\le \acute{k}$. This further implies that the instability condition depends on the sign of the product *PQ*. Therefore, the IAW becomes modulationally stable ($\acute{\omega }^{2}>0$) when the product “*PQ*” is less than zero. This modulationally stable wave propagates as a dark (sometimes known as “black” or “gray”) envelope solitary wave (SW). When the product $PQ>0$, then $\acute{\omega }^{2}<0$, and the waves become modulationally unstable. The modulationally unstable wave packet may propagate as a bright envelope solitary wave, rogue waves (RWs) such as Peregrine solitons (first-order RWs) and super RWs, and breathers such as Akhmediev breathers (spatially periodic wave) or Kuznetsov-Ma solitons (temporally periodic wave)^55–59^. For the unstable case, we may attain the expression of the growth rate of instability $\Gamma =\operatorname {Im}(\acute{\omega })$ as

24 $$\begin{aligned} \Gamma =P\acute{k}^{2}\sqrt{\frac{k_{c}^{2}}{\acute{k}^{2}}-1}. \end{aligned}$$

As the instability relies on the sign of the product *PQ*, it can be numerically investigated by considering the exact parameters of a plasma model.The relativistically degenerate electron-positron plasma is very dense and abundantly found in neutron stars, pulsars, black holes, active galactic nuclei, and accretion disks. We here assume the plasma system in the vicinity of palsars. Currently, the EP pair plasma may be magnetized in the pulsar magnetosphere; however, that of the early universe is likely to be unmagnetized^47^. The densities at which the degenerate electrons and positrons behave nonrelativistically or relativistically are determined by Chandrasekhar’s relativistic parameter $\eta _{j}=p_{Fj} /m_{e}c=\left( n_{j}/n_{c}\right) ^{1/3}$, where $n_{c}\simeq 5.9\times 10^{29}cm^{-3}$. The electrons or positrons become relativistically degenerate for $\eta _{j}>1$. Therefore, when the electrons or positrons lie in the range $n_{eo}$ & $n_{po}\sim 10^{26}cm^{-3}-5.9\times 10^{29}cm^{-3}$, they become nonrelativistic. On the other hand, both electrons and positrons become relativistically degenerate when $n_{e0}$ & $n_{p0}>5.9\times 10^{29}cm^{-3}$. The relativistically degenerate plasma becomes ultrarelativistic for much higher densities, i.e., $n_{e0}$ & $n_{p0}>10^{32}cm^{-3}$^51^ .Here, it is important to mention that the annihilation time $\tau _{ann}$ of electrons and positrons is much greater than the inverse plasma frequency of electrons and positrons $\omega _{pj}(\equiv \sqrt{4\pi n_{e0}e^{2}/m_{e}})$ for these densities. The annihilation time of a particle depends upon the cross section of annihilation, which decreases at high energies and varies for nonrelativistic and ultrarelativistic energies^13^. The annihilation time for electron-positron pair turns out to be of the order of $10^{-15}$s and $10^{-18}$s for nonrelativistic and ultrarelativistic cases, respectively^8^. On the other hand, the inverse plasma frequency for these high density plasmas turns out to be as low as $10^{-19}$s for nonrelativistic case and $10^{-21}$s for ultrarelativistic case. Therefore, the wave propagates in a time much shorter than the annihilation time of electrons and positrons, and hence the pair annihilation is ignored, and the wave is investigated for relativistically degenerate EPI plasmas^8,13^.Furthermore, most astrophysical plasmas contain ions along with electrons and positrons. The properties of the IAWs in the resultant plasma differ from the electron-positron pair plasmas and the ordinary electron-ion plasmas. The reason is that in pure IAW, the oscillation arises due to the charge separation, and electrons provide the restoring force due to the large inertia of ions. However, in pure electron-positron plasmas, charge separation cannot play a vital role in structure formation. For the case of EPI plasma, the situation changes, although the charge separation is still small due to the tiny minority of ion particles. In plasma systems, such as pulsars, there is a scant amount of ions compared to the number of electrons and positrons. The positron-to- electron ratio $\alpha$ is, therefore, taken to be $\alpha =0.6$, 0.7, 0.8, etc., for the numerical analysis to ensure the charge-neutrality condition.

### MI for nonrelativistic electrons and positrons

**Fig. 1:**
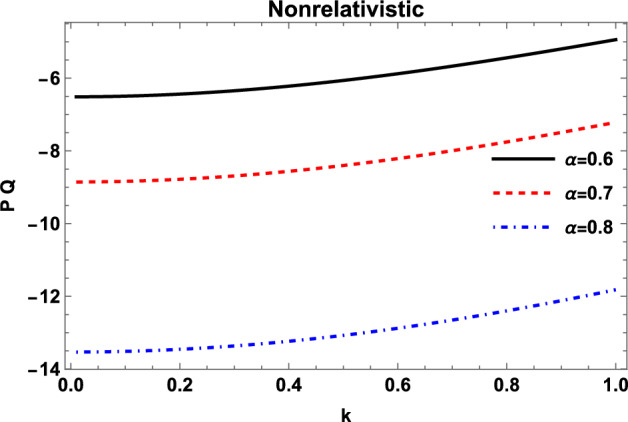
Variation of the nonrelativistic coefficients product (*PQ*) with wave number *k* for different values of density ratio of positrons to electrons $\alpha$ with $n_{p0} =10^{28}cm^{-3}$.

We first consider the case of nonrelativistic dense plasma, where the density of positrons is assumed to be of the order of $10^{28}cm^{-3}$. The density of the electrons is evaluated by taking $\alpha =0.6$. The quantum effects become considerable for these values of densities, since the Fermi length $\lambda _{Fj}=(2k_{B}T_{Fe}/4\pi n_{j0}e^{2})^{1/2}$ of electrons and positrons becomes comparable to their mean interparticle distance, approximated by the Wigner-Seitz radius $d_{j}=(3/4\pi n_{j0})^{1/3}$. The Coulomb coupling parameter $\Gamma _{j}=e^{2}/k_{B}T_{Fj}d_{j}$ remains less than one for ions ($H^{+}$), electrons, and positrons, so we may ignore the correlations (e.g., ion crystallization) among them and assume the fluid model for EPI plasma. At the density of $10^{28}cm^{-3}$, the value of the Fermi temperature comes out to be $T_{Fj}\sim 10^{8}K$ and, therefore, electrons and positrons can be taken to be nonrelativistic ($\eta _{j}<1$) and fully degenerate ($T_{Fj}\gg T$), where the plasma temperature $T\sim 10^{6}K$. The Fermi temperature of the ions remains less than the system temperature, so that the ions behave nondegenerately.The variation of the product *PQ* for the nonrelativistic limit is shown in Fig. 1, which depicts that the product *PQ* remains negative for all values of the normalized propagation vector *k*.

This shows that the system remains modulationally stable for all propagation vectors for the nonrelativistically degenerate EPI plasmas. The behavior is further analyzed for different values of the ratio of the number of positrons to electrons $\alpha$. The figure shows that the product *PQ* experiences a decrement with the increasing number of positrons in the system which suggests that the enhancement in the concentration of positrons augments the stability of the IAW packets in EPI plasma.

### MI for ultrarelativistic electrons and positrons

**Fig. 2:**
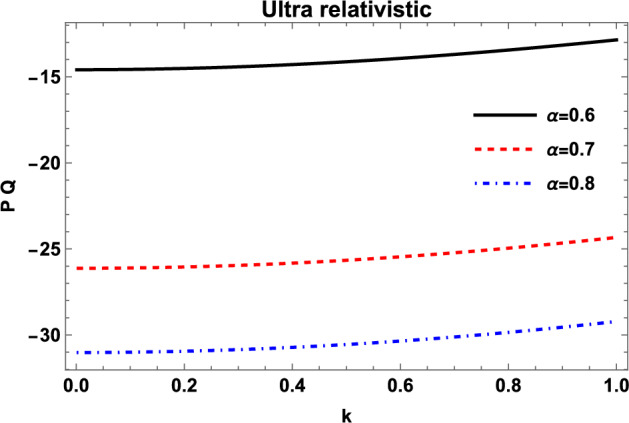
Variation of the ultrarelativistic coefficients product (*PQ*) with wave number *k* for different values of density ratio of positrons to electrons $\alpha$ with $n_{p0}=10^{32}cm^{-3}$.

We may compare the graphs of *PQ* for nonrelativistic plasma with that of the ultrarelativistically degenerate EPI plasma. Here, we assume the density of ultrarelativistic electrons and positrons to be of the order of $10^{32} cm^{-3}$ for which the Fermi energy of electrons and positrons becomes greater than their respective rest mass energies. The ion crystallization effects are again ignored as the Coulomb coupling parameter $\Gamma _{i}$ is less than one. The instability analysis of the ultrarelativistic EPI plasma through the product *PQ* is shown in Fig. 2.

The figure dictates that, for the case of ultrarelativistic EPI plasma, the waves remain modulationally stable for the whole range of the propagation vector *k*. Furthermore, similar to the nonrelativistic case, the increasing parameter $\alpha$ stabilizes the system of ultrarelativistically degenerate EPI plasma. As mentioned above, we get modulationally stable waves for both nonrelativistic and the ultrarelativistic cases of EPI plasmas. However, it is important to mention that the nonlinearity coefficient *Q* remains positive for all ranges of plasma parameters, and the negative product *PQ* arises due to the negative value of the dispersion coefficient *P*. This situation in a plasma system is similar to that of the optical solitons in optical fibers, which results in the dark envelope solitons. In the next section, we shall discuss the envelope solitary solutions of the system under consideration.

## Exact dark soliton solutions

Modulationally stable (unstable) wave packets result in dark (bright) envelope solitons. For the case of nonrelativistic and ultrarelativistic degenerate EPI plasmas, the system remains modulationally stable for all the related plasma parameters.

**Fig. 3:**
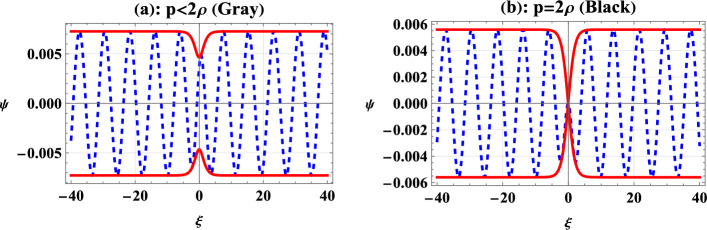
Classification of dark envelope solitons as gray and black envelope solitons. Here $p=1$, $k=0.9$, $\tau =1$, $k_{1}=0.8$ while the vacuum solution has $\rho =0.65$ for gray envelope soliton and $\rho =0.5$ for black envelope soliton.

Therefore, only dark-modulated envelope solitons are admissible for these plasmas. The dark solitons are characterized as a localized depletion of amplitude on a constant background of a wave train that stays consistent on both sides of the soliton. Therefore, the wave packet appears as a propagating localized hole or void inside an area of uniform wave energy. These structures are further classified as black and gray modulated envelope solitons depending upon the relation of the amplitude of the wave train with the propagation vector of the dark solitons.

### Hirota formalism

The dark soliton solutions of the NLS Eq. (20) can be evaluated by many methods, such as the inverse scattering transform, Darboux transformation, the Hirota method, Bäcklund transformation, etc.^60^. In this investigation, we use the Hirota method^48^, a systematic and direct method, where we transform the NLPDE into a bilinear form as follows:

25 $$\begin{aligned} \left. P\left( \frac{\partial }{\partial t}-\frac{\partial }{\partial \acute{t}},\frac{\partial }{\partial x}-\frac{\partial }{\partial \acute{x}}\right) f(x,t).g(\acute{x},\acute{t})\right| _{t=\acute{t},x=\acute{x} }=0. \end{aligned}$$

The bilinear equation is converted into Hirota bilinear equation in terms of the following Hirota operators

26 $$\begin{aligned} D_{t}^{m}D_{x}^{n}f.g=\left. \left( \frac{\partial }{\partial t} -\frac{\partial }{\partial \acute{t}}\right) ^{m}\left( \frac{\partial }{\partial x}-\frac{\partial }{\partial \acute{x}}\right) ^{n}f(x,t).g(\acute{x},\acute{t})\right| _{t=\acute{t},x=\acute{x}} \end{aligned}$$

and the exact solutions can be obtained using the perturbation approach. To find the dark envelope solution of the NLS Eq. (20) with normal dispersion (negative *P*), the following transformation is assumed

27 $$\begin{aligned} \Psi =\sqrt{-\frac{2P}{Q}}\frac{g}{f}, \end{aligned}$$

where *g* is a complex function and both *f* and *g* are the dependent variable functions. Using Eq. (27) in Eq. (20) gives the following coupled equations in terms of Hirota operators

28 $$\begin{aligned} \left\{ \begin{array}{c} \left. \left( iD_{\tau }+PD_{\xi }^{2}-\lambda \right) \left( g.f\right) =0,\right. \\ \left. \left( PD_{\xi }^{2}-\lambda \right) (f.f)=2P\left| g\right| ^{2}.\right. \end{array} \right. \end{aligned}$$

Here, $\lambda$ is to be determined. Using the perturbation series for the functions $\left( f,g\right)$, we may expand *f* and *g* as follows

29 $$\begin{aligned} f&=1+\epsilon f_{1}+\epsilon ^{2}f_{2}+...,\nonumber \\ g&=g_{0}(1+\epsilon g_{1}+\epsilon ^{2}g_{2}+...), \end{aligned}$$

where $g_{0}$ is called the vacuum solution used to demonstrate the dark solitons over a background wave train. We assume it to be of the form $g_{0}=\rho \exp [i(k_{1}\xi +\omega _{1}\tau )]$ that accounts for the fulfillment of the boundary condition $\left| g_{0}\right| ^{2}=\rho ^{2}$ as $\xi \rightarrow \pm \infty$. Substituting the function expansions (29) into Eq. (28), we get the equations for various orders of $\epsilon$. Considering first the zeroth-order of $\epsilon$ equations, we obtain the following expressions

30 $$\begin{aligned} \left\{ \begin{array}{c} \lambda =-2P\rho ^{2}\text {, }\\ \omega _{1}=-P(k_{1}^{2}+2\rho ^{2}), \end{array} \right. \end{aligned}$$

which gives the vacuum or the background wave train solution as follows

$$\begin{aligned} g_{0}=\rho \exp [i(k_{1}\xi -P(k_{1}^{2}+2\rho ^{2})\tau )]. \end{aligned}$$

### Single dark soliton solution

In order to find one-envelope hole soliton solution of the given NLPDE, we take

31 $$\begin{aligned} \left\{ \begin{array}{c} f_{1}=\exp [\chi ],\\ g_{1}=Z\exp [\chi ], \end{array} \right. \end{aligned}$$

where *Z* is a complex constant and $\chi =p\xi +\Omega \tau$.

The advantage of exponential functions is that the infinite perturbative expansion of *f* and *g* is truncated to $\varepsilon -$order for single soliton. Here, *p* is the constant propagation vector, and $\Omega$ is the frequency of the soliton. The $\varepsilon -$order of Eq. (28) gives the linear dispersion relation and the complex constant, respectively, as follows

32 $$\begin{aligned} \left\{ \begin{array}{c} \Omega =-Pp\left( 2k-\sqrt{4\rho ^{2}-p^{2}}\right) ,\\ \,Z=\frac{\sqrt{4\rho ^{2}-p^{2}}+ip}{\sqrt{4\rho ^{2}-p^{2}}-ip}. \end{array} \right. . \end{aligned}$$

The single dark envelope soliton solution, therefore, reads as follows

33 $$\begin{aligned} \Psi =\sqrt{-\frac{2P}{Q}}\frac{\rho \exp \left[ i(k_{1}\xi +\omega _{1} \tau )\right] (1+Ze^{\chi })}{(1+e^{\chi })}. \end{aligned}$$

It is important to mention that this solution is applicable when the dispersion *P* is negative, which gives the positive values of the physical parameters (propagation vector, frequency, etc.) and the propagation of the dark envelope soliton towards the left.

It can be seen from Eq. (32) that the real value of frequency $\Omega$ is only attained for $p^{2}\le (2\rho )^{2}$ or $p\le (2\rho )$. This classifies the dark-envelope solitons into two further classes: $p<2\rho ,$ called the gray-envelope soliton and $p=2\rho ,$ called the black soliton. The difference between both hole envelope solitons is graphically shown in Fig. 3. In this figure, the real part of the solution $\Psi$, plotted as a dashed curve, shows a dip or a hole of a dark soliton on the background wave train $g_{0}$. The absolute values are plotted as solid curves to compare the relative depth of single gray and black solitons in Fig. 3a,b, which show that the black soliton allows the deepest dip on the background wave train.

**Fig. 4:**
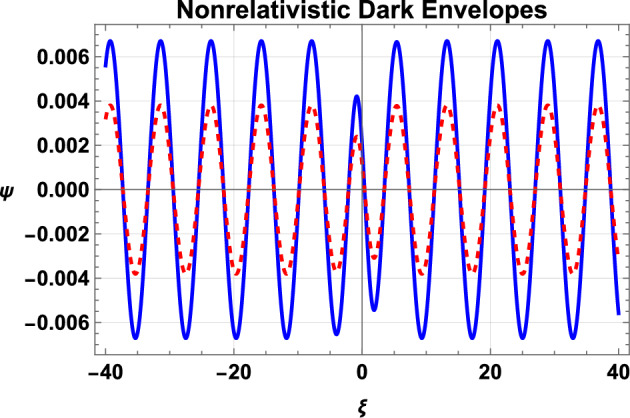
Variation of dark IA envelope soliton as given by Eq. (33) for nonrelativistic EPI plasma with the density ratio $\alpha$. Here, the solid curve is for $\alpha =0.8$, and the dashed curve is plotted for $\alpha =0.85$. Remaining parameters are $n_{p0}=10^{28}cm^{-3}$, $p=1$, $\rho =0.6$, $\tau =0$, $k=0.9$, and $k_{1}=0.8$.

**Fig. 5:**
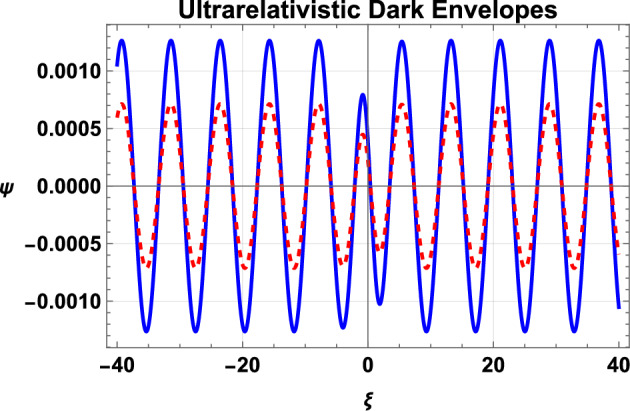
Variation of dark IA envelope soliton as given by Eq. (33) for ultrarelativistic EPI plasma with the density ratio $\alpha$. Here, the solid curve is for $\alpha =0.8$, and the dashed curve is plotted for $\alpha =0.85$. Remaining parameters are $n_{p0}=10^{32}cm^{-3}$, $p=1$, $\rho =0.6$, $\tau =0$, $k=0.9$, and $k_{1}=0.8$.

We have also shown the effects of the positron concentration $\alpha$ on the single dark-envelope IA soliton for nonrelativistic and ultrarelativistic cases of electrons and positrons. Figure 4 shows the effect of the positron concentration $\alpha$ on the dark IA envelope soliton with nonrelativistic electrons and positrons. The figure shows that the increase of the positron concentration $\alpha$ tends to reduce the amplitude of the dark soliton. A similar behavior is shown for the case of ultrarelativistic electrons and positrons, as depicted in Fig. 5. Moreover, the comparison of Figs. 4 and 5 show that the amplitude of the dark envelope solitons with nonrelativistic positrons and electrons is higher than that of the dark solitons with ultrarelativistic electrons and positrons. The reason is that with an increase in the ratio $\alpha$ or the density of positrons/electrons, the density of ions reduces and, therefore, the amplitude of the IAW manifests a mitigation.

### Interaction of dark solitons

The overtaking interaction of the dark envelope solitons is studied by evaluating the two soliton solutions of the NLS Eq. (20). It is important to mention that for dark multisoliton solutions, each dark soliton has to be observed on the same background wave train. Therefore, the frequency and amplitude of the vacuum $g_{0}$ are global parameters. For a two-soliton solution, we start with the following pair of functions

34 $$\begin{aligned} \left\{ \begin{array}{c} f_{1}=\exp [\chi _{1}]+\exp [\chi _{2}]\text {,}\\ g_{1}=Z_{1}\exp [\chi _{1}]+Z_{2}\exp [\chi _{2}], \end{array} \right. \end{aligned}$$

where $$\chi _{j}=p_{j}\xi +\Omega _{j}\tau$$, and $$j=1,2$$.

The dispersion relation $\Omega _{j}$ is determined from the $\varepsilon -$order of Eq. (28) as follows

35 $$\begin{aligned} \Omega _{j}=-Pp_{j}(2k-\sqrt{4\rho ^{2}-p_{j}^{2}}) \& Z_{j} =\exp [2i\theta _{j}]. \end{aligned}$$

Here, $p_{j}$ are the constant propagation vectors satisfying $p_{j}\le 2\rho$ and $\theta _{j}=\sin ^{-1}(p_{j}/2\rho ).$

Next, we assume the functions

36 $$\begin{aligned} \left\{ \begin{array}{c} f_{2}=Y_{12}\exp [\chi _{1}+\chi _{2}],\\ g_{2}=Z_{12}\exp [\chi _{1}+\chi _{2}] \end{array} \right. \end{aligned}$$

and substitute Eq. (36) into Eq. (28) which truncate the higher-order terms, and then the expressions of interaction parameters $Y_{12}$ and $Z_{12}$ are obtained from the $\varepsilon ^{2}-$order equations of Eq. (28) as follows:

37 $$\begin{aligned} Y_{12}=\left[ \frac{\sin (\theta _{1}-\theta _{2})}{\sin (\theta _{1}+\theta _{2} )}\right] ^{2}\ \text {and }Z_{12}=Z_{1}Z_{2}Y_{12}. \end{aligned}$$

The exact two dark envelope soliton solution of the NLS Eq. (20) reads as

38 $$\begin{aligned} \Psi =\sqrt{-\frac{2P}{Q}}\frac{\rho \exp \left[ i(k_{1}\xi +\omega _{1} \tau )\right] (1+Z_{1}e^{\chi _{1}}+Z_{2}e^{\chi _{2}}+Z_{12}e^{\chi _{1} +\chi _{2}})}{(1+e^{\chi _{1}}+e^{\chi _{2}}+Y_{12}e^{\chi _{1}+\chi _{2}} )}. \end{aligned}$$

**Fig. 6:**
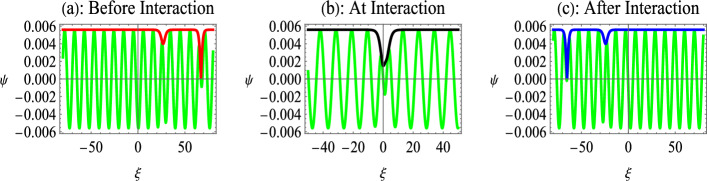
Interaction of black and gray envelope solitons for nonrelativistic EPI plasma given by Eq. (38). Here, $\tau =-2000$ before interaction in (**a**), $\tau =0$ at interaction point in (**b**), and $\tau =2000$ after interaction in (**c**). Other parameters are $n_{p0}=10^{28}cm^{-3}$, $\alpha =0.8$, $p_{1}=1$, $p_{2}=0.7$, $\rho =0.5$, $k=0.9$, and $k_{1}=0.6$.

This solution represents the two solitons propagating towards the left, where the coefficients *P* and$\ Q$ depend on the density of relativistically degenerate electrons and positrons. It is important to mention that the interaction can only take place between a black and a gray soliton or between two gray solitons. The interaction between black solitons is not possible since the overtaking interaction can only take place when the amplitude/propagation vectors of interacting solitons are different, i.e., $p_{1}\ne p_{2}$. The background parameters are global for both solitons, i.e., $\rho$ is fixed for both solitons, so that both black solitons would have the same propagation vector determined by $p_{j}=2\rho$.

**Fig. 7:**
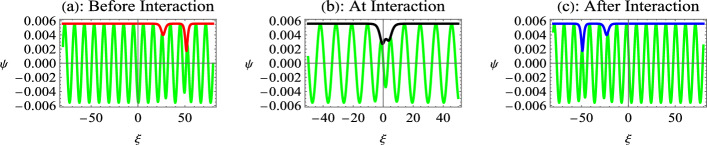
Interaction of two gray envelope solitons for nonrelativistic EPI plasma given by Eq. (38). Here, $p_{1}=0.95$, $p_{2}=0.7$, $\rho =0.5$, and remaining parameters are same as in Fig. 6.

We first study the interaction of a black ($p_{1}=2\rho$) and a gray ($p_{2}<2\rho$) soliton for nonrelativistic electrons and positrons in Fig. 6. Figure 6a shows the plot of $\operatorname {Re}[\Psi ]$ and $\ Abs[\Psi ]$ for the time before interaction, which is conventionally taken to be negative. It depicts the propagation of two dark envelope solitons towards the left, where the taller black soliton propagates towards a shorter gray soliton. At the point of interaction (Fig. 6b), the two dark-envelope solitons merge into a single dark-envelope soliton. The amplitude of the dark envelope soliton at the interaction point is lower than the sum of the amplitudes of the individual solitons before and after the interaction. This shows that at the interaction point, the nonlinear superposition of dark envelope solitons takes place like the KdV-type solitons^25,61^. Figure 6c indicates that the taller black envelope soliton overtakes the shorter gray envelope soliton after interaction without changing shape. Therefore, the two-soliton interactions of the dark envelopes indicate that the interaction is similar to the interaction of KdV-type solitons.

**Fig. 8:**
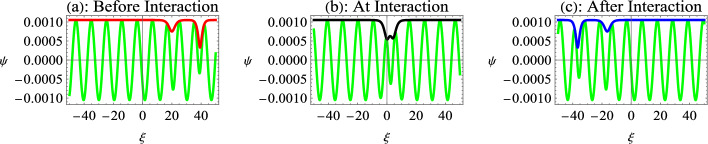
Interaction of two gray envelope solitons for ultrarelativistic EPI plasma. Here, $\tau =-5000$ before interaction in Fig 7a, $\tau =0$ at interaction point in Fig 7b, and $\tau =5000$ after interaction in Fig 7c. Other parameters are $n_{p0}=10^{32}cm^{-3}$, $\alpha =0.8$, $p_{1}=0.95$, $p_{2}=0.7$, $\rho =0.5$, $k=0.9$, and $k_{1}=0.8$.

Figure 7 indicates the interaction of gray-gray envelope solitons, where $p_{1}=0.95$, $p_{2}=0.7$ and $\rho =0.5$. The comparison of the graphs in Figs. ﻿6 and 7 shows that with a small change in the value of $p_{1}$ (from 1 to 0.95), the spatial scale of the interaction reduces for the gray-gray interaction compared to the black-gray interaction. This is equivalent to an increase of the temporal scale for gray-gray interaction, showing that the gray envelope soliton takes more time to interact with a gray soliton, as compared to a black soliton. This happens because the shorter solitons travel slowly and take a longer time to interact. Therefore, a black-gray interaction is a faster interaction compared to the gray-gray interaction. Furthermore, the composite structure at the point of interaction becomes a two-dip soliton compared to a single-dip dark soliton for the case of black-gray interaction.

Figure 8 further shows the interaction of two gray solitons for the case of ultrarelativistically degenerate electrons and positrons. The plots again show the nonlinear superposition of dark-envelope solitons in ultrarelativistically degenerate EPI plasma. The spatial region of the soliton interaction reduces, or equivalently, the temporal regime of the soliton interaction increases for ultrarelativistic EPI plasma as compared to the nonrelativistic EPI plasma. This happens as the soliton amplitude reduces for the ultrarelativistic plasma compared to the nonrelativistic plasma, which reduces the speed of solitons; hence, the solitons take more time to show interaction.

## Discussion and conclusion

We have studied the IAWs in an electron-positron-ion (EPI) plasma with relativistically degenerate electrons and positrons usually found near pulsars. For the EPI system, the nonlinear Schrödinger (NLS) equation has been obtained by applying the derivative expansion method, and a condition for modulational stability/instability has been determined for the system. It has been observed that the EPI system remains modulationally stable for the plasma parameters of nonrelativistic and ultrarelativistic EPI plasmas for all values of the propagation vector. Furthermore, the increase in positron concentration has been shown to augment the stability of the nonlinear ion acoustic wave packets both in nonrelativistic and ultrarelativistic EPI plasmas. The dark envelope soliton solutions have been obtained for the modulationally stable system using Hirota’s bilinear method. These envelopes, characterized as a void/hole over a background wave train, have been further classified as black and gray solitons depending upon the relation between the background wave train’s amplitude and the envelope soliton’s propagation vector. The increase in the positron concentration has been shown to reduce the amplitude of the dark envelope soliton. Furthermore, the amplitude of the envelope soliton has been found to mitigate more significantly for ultrarelativistic EPI plasmas as compared to their nonrelativistic counterparts.

The two dark-envelope soliton solution has also been obtained, and it has been found that the two black solitons cannot undergo overtaking interaction. The interaction of black and gray, and the two gray solitons has been discussed for non/ultrarelativistic degenerate EPI plasma. It has been found that the dark envelope solitary waves interact nonlinearly, similar to the KdV-type solitons, so the condition of linear superposition is not met at the interaction point. The spatial regime of the soliton interaction has been observed to be reduced (or, equivalently, the temporal region of the interaction has increased) for the gray-gray envelope interaction compared to the black-gray envelope interaction. Furthermore, the temporal regime of soliton interaction has been shown to increase for ultrarelativistic EPI plasma by comparison with its nonrelativistic counterpart, as the shorter solitons travel with a slower speed and take a longer time to interact. The present model of dark soliton interaction is not only applicable to dense astrophysical plasmas but also to space and laboratory plasma systems, and a variety of physical situations of interest, such as optical fibers, Madelung fluid, and Bose-Einstein condensates, where the dark solitons have been observed, with suitable adjustments. This paper embarks on a new path of interaction of NLS solitons in plasmas, which hopefully will open new avenues of research for scientists working in this field and also encourage the researchers in other disciplines of physics to pursue this area of study and develop physical models relevant to their research domains.

**Future work:** This study examines the overtaking collision of two modulated envelope dark solitons utilizing the Hirota Method. The overtaking collision is recognized to occur when the waves propagate in the same direction, specifically when the angle between the trajectories of the two solitons is zero. Nevertheless, when the two solitons collide head-on (face-to-face), i.e., at an angle of 180 degrees, Hirota’s technique becomes invalid. Instead, the extended Poincaré-Lighthill-Kuo (PLK) method is employed to examine head-on collisions^62–65^. In this instance, the velocities of the colliding solitons may not necessarily vary, yet they can assume any magnitude. This is regarded as one of the most pivotal future endeavors for investigating modulated envelope dark soliton collisions^66^ in the current plasma model. Furthermore, in future research, we will investigate the impact of fractionality on the dynamics of modulated envelope dark solitons by analyzing the following time fractional planar NLS equation and deriving analytical solutions for the fractional modulated envelope dark solitons utilizing more precise mathematical techniques, including the residual power series method, the new iterative method^67–69^, the Tantawy technique^70–73^, among others:

$$\begin{aligned} iD_{\tau }^{\beta }\Psi +P\partial _{\xi }^{2}\Psi +Q\Psi \left| \Psi \right| ^{2}=0,\, \forall \,0<\beta \le 1, \end{aligned}$$

where $D_{\tau }^{\beta }\equiv \partial ^{\beta }/\tau ^{\beta }$ indicates the fractional Caputo derivative operator of order $\beta$.

For the investigation of fractional envelope dark solitons, the subsequent initial condition is considered: where *Z* is a complex constant, and $\chi =p\xi +\Omega \tau$.

$$\begin{aligned} \mathbf {\Psi =}\sqrt{-\frac{2P}{Q}}\frac{\rho \exp \left[ i(k_{1}\xi )\right] (1+Ze^{p\xi })}{(1+e^{p\xi })}\mathbf {.} \end{aligned}$$

## Data Availability

The datasets used and/or analyzed during the current study are available from the corresponding author on reasonable request.

## Declarations

### Use of artificial intelligence (AI) tools declaration

The authors declare that they have not used AI tools in the creation of this article.
